# Implementing a One Health approach to emerging infectious disease: reflections on the socio-political, ethical and legal dimensions

**DOI:** 10.1186/s12889-015-2617-1

**Published:** 2015-12-29

**Authors:** Chris Degeling, Jane Johnson, Ian Kerridge, Andrew Wilson, Michael Ward, Cameron Stewart, Gwendolyn Gilbert

**Affiliations:** Centre for Values, Ethics and the Law in Medicine, K25 Level 1, Medical Foundation Building, University of Sydney, Sydney, NSW 2006 Australia; Marie Bashir Institute for Infectious Disease and Biosecurity, University of Sydney, Sydney, Australia; Faculty of Veterinary Medicine, University of Sydney, Sydney, Australia; Menzies Centre for Health Policy, University of Sydney, Sydney, Australia; Sydney Law School, University of Sydney, Sydney, Australia; Centre for Infectious Disease and Microbiology – Public Health, Westmead Hospital, Sydney, Australia

**Keywords:** One health, Emerging infectious disease, Zoonoses, Bioethics, Health policy, Health law

## Abstract

**Background:**

‘One Health’ represents a call for health researchers and practitioners at the human, animal and environmental interfaces to work together to mitigate the risks of emerging and re-emerging infectious diseases (EIDs). A One Health approach emphasizing inter-disciplinary co-operation is increasingly seen as necessary for effective EID control and prevention. There are, however, socio-political, ethical and legal challenges, which must be met by such a One Health approach.

**Discussion:**

Based on the philosophical review and critical analysis of scholarship around the theory and practice of One Health it is clear that EID events are not simply about pathogens jumping species barriers; they are comprised of complex and contingent sets of relations that involve socioeconomic and socio-political drivers and consequences with the latter extending beyond the impact of the disease. Therefore, the effectiveness of policies based on One Health depends on their implementation and alignment with or modification of public values.

**Summary:**

Despite its strong motivating rationale, implementing a One Health approach in an integrated and considered manner can be challenging, especially in the face of a perceived crisis. The effective control and prevention of EIDs therefore requires: (i) social science research to improve understanding of how EID threats and responses play out; (ii) the development of an analytic framework that catalogues case experiences with EIDs, reflects their dynamic nature and promotes inter-sectoral collaboration and knowledge synthesis; (iii) genuine public engagement processes that promote transparency, education and capture people’s preferences; (iv) a set of practical principles and values that integrate ethics into decision-making procedures, against which policies and public health responses can be assessed; (v) integration of the analytic framework and the statement of principles and values outlined above; and (vi) a focus on genuine reform rather than rhetoric.

## Background

The recent Ebolavirus (EBOV) outbreak in West Africa and continuing human infections with a novel H7N9 influenza A virus in mainland China are salient reminders of how human and nonhuman health are inextricably linked. Nonhuman animals are the source of 70 % of emerging and re-emerging infectious disease (EID) threats to human health [1], and more than half of all established human pathogens [2]. The threats posed by EIDs are dynamic. EIDs are caused by pathogens that can change their behaviour over time – either through genetic modification or through changes in the patterns and pathways of transmission [3]. Social, economic and political systems can either promote or inhibit pathogen transfer, and the incidence and pathogenicity of the disease [4]. While a lack of data makes quantitation difficult, EIDs and zoonoses account for a significant proportion of the global disease burden [5]. EIDs and emergence of zoonotic pathogens, including human immunodeficiency virus (HIV), are direct causes of an estimated 15 million deaths worldwide each year [6].

A One Health approach is increasingly considered to be the most effective way of managing EID threats [7, 8] because it represents an acknowledgement of certain facts about the nature of disease, which are then deployed to structure the response. One Health is grounded in a recognition that human, animal and environmental health are interdependent [9], that animal species provide a shared reservoir for pathogen exchange and spread, and that many EIDs are driven by varied and dynamic human-animal interactions [9, 10]. The response One Health offers is to deconstruct the disciplinary silos [11] which have separated biomedical and social sciences devoted to the study of human disease from those devoted to nonhuman disease and ecological concerns [12, 13]. Inter-disciplinary research is called for and required, as is interventionist practice at local, national and international levels involving: policymakers, planners, regulators, physicians, veterinarians, ecologists, public and animal health officials, environmental health officers, microbiologists, and other allied natural and social scientists [10, 14].

Although principally associated with EID prevention and control, One Health is also relevant to prevention and control of endemic and zoonotic animal diseases, as well as securing food safety [15, 16]. Considering the magnitude and complexity of global issues surrounding infectious disease and food security, the One Health approach has the potential to provide the creative, effective and sustainable solutions required.

Despite its strong motivating rationale, implementing a One Health approach can be challenging. Dealing with EIDs in an integrated and considered manner can be highly problematic, especially in the face of a perceived crisis. In this paper we examine the socio-political, ethical and legal considerations implied by a One Health approach to EIDs. First we describe how a One Health approach could galvanise and enhance current capacity in EID prevention and control. Making reference to case examples, we then identify and characterise socio-political, ethical and legal concerns that have the potential to limit the effectiveness of One Health interventions. Finally, we draw on this data to provide guidance as to how these concerns and issues might be addressed, and point to remaining challenges to the likely success of the One Health approach to EID control and prevention.

## Methods

In order to explore the broader implications of a One Health approach we employed philosophical and qualitative methods to map existing and potential scientific, ethical and political responses to EIDs in Australia and our region. The overarching philosophical approach is that of developing sustained arguments that critically analyse the existing literature and reconceptualise or refine key concepts. This conceptual information is often observed in exemplars and paradigm cases. In particular we focused on materials pertaining to the social, political and ethical consequences of responses to the risks posed to human health and wellbeing by Hendra virus [HeV], Nipah virus [NiV] and Rabies virus [RbV] in Australasia, and compared them with international responses to canonical examples of pandemic and food borne zoonoses – severe acute respiratory syndrome (SARS) [17] and bovine spongiform encephalitis/variant Creutzfeldt Jacob disease (BSE/vCJD), respectively. A synopsis of the characteristics and burdens of these diseases and the pathogens that cause them are outlined in Boxes 1 and 2.

Because our aim was to generate inductive insights and develop a robust set of arguments – rather than a comprehensive catalogue of every case example or publication – the sample evolved iteratively from searches of textual sources such as publicly available international (e.g. WHO) and government reports; academic databases (e.g. PubMed); online/print news services (Factiva); organizational newsfeeds (Centers for Disease Control); and the websites of major One Health collaborations [18]. Materials in the sample were read and qualitatively reviewed through an iterative process of testing, revising and refining our definitions, principles and theoretical generalisations [19, 20] – against the emerging conceptual map and feedback from the research team. Led by the first author, this cycle of searching, mapping and critical analysis continued until a period where new textual materials were not providing substantive new insights and the team was confident that a position of conceptual saturation had been achieved. In what follows we draw on these analyses and reflections to describe the content, context and nature of the challenges that need to be faced for the effective implementation of a One Health approach to EID control and prevention.

### Findings

#### EID prevention and control strategies require a One Health approach

One Health is a holistic approach that emphasizes, but is not restricted to, the need to understand and regulate the environmental context (human-animal-ecosystem interface) of disease emergence and expression [21]. EIDs are characterized by their complexity and uncertainty as to their causes, consequences and likely solutions [22]. In broad terms, the occurrence and cross-species transmissibility of many emerging pathogens, like Ebolavirus (EBOV) and H7N9, arise from human activities such as changes in land use, growth in global trade and travel and intensification of animal husbandry practices [23–25]. The speed with which our understanding of the biology and epidemiology of H7N9 has developed demonstrates how much our ability to respond to new EID threats has improved over the last few decades. Yet despite advances in immunobiology and genomics that have contributed to diagnostics, therapeutics, and vaccine development, the threat of EIDs to human health and community wellbeing persists.

Part of the reason why EID threats remain in spite of scientific advances, are that EID events are not simply about pathogens jumping species barriers. The threats posed by EIDs are comprised of complex and contingent sets of relations that involve socioeconomic and socio-political drivers and consequences, with the latter extending beyond the impact of the disease. The social, cultural and economic impacts of zoonoses are significant. The examples contained in Tables 1 and 2 demonstrate the difficult balance between the human health risks and socioeconomic and cultural costs of EID control [26, 27]. Policy decisions should be based on sound evidence – but it is often the case in dealing with EIDs that the evidence required is absent or fluid. EID events are often dynamic situations that are characterised by uncertainty. As events unfold new evidence is created. Consequently decisions made on the basis of present data can be seen as wrong in the future, as more evidence and a better understanding emerges.

**Table 1:** EIDs of immediate importance to Australasia

*Hendra virus* infection is endemic among at least two species of flying fox in Australia and causes rare, but catastrophic, human infection [85]. Loss of habitat has led to increasingly intense incursions of flying foxes into populated rural and peri-urban areas and promoted the ‘spill-over’ of Hendra virus into horses and then to people [86]. Hundreds of people have been directly exposed to Hendra virus, with seven confirmed human infections and four deaths since 1994. With over one hundred dead horses and persistent risk, the emergence of Hendra has had significant impact on equine and tourist industries in north eastern Australia, diverted major research resources and caused significant distress and controversy in the broader community [31, 87].
*Nipah virus*, a close relative of Hendra, is endemic in East Asian flying fox populations. In 1999, after a program of deforestation and agricultural development in Eastern Malaysia it spread to pigs then humans and other animals, causing respiratory disease and severe encephalitis [88]. It subsequently was reported in India and Bangladesh. Humans can be infected directly from bats, by ingestion of contaminated food and from other humans. Among 522 confirmed human cases, the overall mortality was greater than 50 % [89]. Nipah control programs devastated Malaysia’s pig industry and caused high unemployment and dislocation of rural populations, at a cost of more than US$1 billion to the national economy [90]. Nipah virus has been identified by WHO as a likely cause of future pandemics.
*Rabies virus* infects the central nervous systems of people, wildlife and domestic mammals. The disease is transmitted by bites from infected animals and once it becomes symptomatic, it is virtually always fatal. 55,000 people die and 7.5 million receive post exposure prophylaxis annually, costing $124 billion [91]. Rabies is endemic in much of South East Asia but its range is expanding. Focusing on Australia, the continent is free from Rabies, but the current expansion of the disease in Indonesia [92] is a genuine threat to northern regions. Although likely controllable in domestic dog populations [93], if Rabies were to become endemic amongst wild or feral animals in this setting, current modelling indicates it would be almost impossible to eradicate [94].

**Table 2:** Significant historical (i.e. effectively eradicated) EIDs

*Severe acute respiratory syndrome* (SARS) is a human respiratory infection, caused by a coronavirus isolated from Chinese horseshoe bats [95]. It was first reported in Asia in 2003 and, within a few months, spread to thirty seven countries in the Americas, Europe and Asia. It affected more than 8000 people and caused 774 deaths, before being successfully eliminated by concerted international efforts. The outbreak and fear that another pandemic could occur are estimated to have cost Canadian and east Asian economies US$200 billion [27].
*Bovine spongiform encephalitis/variant Creutzfeldt Jacob disease* (BSE/vCJD) is a rare but fatal human neurodegenerative condition, caused by consumption of bovine products contaminated with the prions that cause BSE. Since vCJD was first identified in 1996, 175 cases have been reported in the UK and forty nine elsewhere. The World Bank estimates that the direct costs of vCJD/BSE to date exceed more than US $11 billion. Infected herds and the control measure imposed to prevent further infections devastated agricultural communities. The impacts of the emergence of a new zoonotic disease amongst the British public were far broader than agriculture, including the cessation of UK plasma production because of potential iatrogenic infection. With an estimated one in 4000 UK residents carrying vCJD, the burdens will continue well into this century [96].

Official reviews of canonical EID events such SARS [17] and BSE/vCJD [28] share two key findings: (i) that actions to reduce risk should not be predicated on scientific certainty; and (ii) that policies to deal with the risks and effects of an EID need to be founded on widely held values, so that people understand, in advance, the kinds of choices that will have to be made. This suggests that the One Health approach needs more than inter-sectoral collaboration and robust health legislation, as **t**he unique nature of EIDs critically limits the effectiveness of scientific, top-down and technocratic approaches to governance [29].

#### Implementing a One Health approach faces socio-political, ethical and legal challenges

The success of One Health depends on more than scientific knowledge and technical achievement because some of the issues that arise in addressing EID risks are so-called ‘wicked problems’ [30]. When a new EID threat emerges there are rarely ready-made solutions and health policymakers and practitioners are often forced to make tragic choices that may contravene widely held values. Considerations must include the need to protect public health and the wider social, economic and environmental impacts of proposed interventions. Economic and political interests can complicate the decision-makers’ motives and decision-maker uncertainty is compounded by policy decisions becoming entangled in political, ethical and legal considerations [31–33]. As events surrounding the EBOV outbreak in West Africa illustrate, the importance placed on a specific EID threat at any one time also depends on who is setting the agenda [34]. Therefore to be successfully implemented, the One Health approach must address a range of socio-political, ethical and legal challenges that arise as a consequence of the spread of infection within and between species. Most of these challenges are not unique to One Health, but are shared by any approach to addressing EIDs. However these challenges frequently go unrecognized. In the following section we will clarify the nature of these issues so they can be addressed later in the paper.

1. **Socio-political challenges**

A focus on individualism, perceptions, short term solutions, populism and avoiding controversy are features of political life, which can prove challenging for EID policy and work against developing effective strategies for addressing EIDs.

Policy responses to EID events such as Nipah and Hendra virus infections (outlined in Box 1) tend to focus on necessary and proximal causes (what individuals do to put themselves at direct risk from an infectious pathogen) because the science about other aspects of EIDs is often complex, uncertain and lacking a clear narrative. Compounding this, our moral psychologies have evolved to respond to direct harms – not indirect distal causal stories. Many people in liberal democracies believe that they are entitled to rights and freedoms that cannot be sacrificed merely for the marginal gains of others. As the discourse surrounding climate change and other wicked problems illustrates, this promotes technological solutions because they do not require substantive changes in human behaviours and underlying values systems. [35] The net result is that the policy focus for EID prevention and control tends to remain on individual behaviours rather than the structural drivers of emergence and transmission – a case example being the focus on vaccine development and the husbandry practices of horse owners in response to the zoonotic risks of Hendra virus [31, 36]..

The political impetus for action in response to many EIDs is not necessarily scientific evidence but societal perceptions. Indeed, in the face of scientific uncertainty and ethical ambiguity, ideological perspectives and short-term political considerations often supplant efforts to devise effective long-term interventions [28, 37].

Political imperatives to avoid, or at least minimise, public concern whilst dealing with EIDs can also prove challenging. In the case of BSE, powerful interests dominated early government responses, leading policymakers to make decisions that avoided public controversy, but had major economic consequences. As the crisis unfolded, expertise became politicized leading to conflict between agencies and policy inconsistency between health communication strategies and the measures being taken to minimize the risks to human health [38]. Even when the link between BSE and vCJD became clear, existing feed bans were poorly enforced and risk communication was dominated by fear of public panic [39]; even as the decision was made to remove all potential sources of human infection from the UK food supply, messages were confused and policy implementation impeded by poor co-ordination between agencies [28].

A common but problematic response to EID threats has been to invoke the precautionary principle. Roughly speaking, the precautionary principle can be applied in situations where human activities create a scientifically plausible, but uncertain, risk of significant harm. In response the principle advocates that actions ought to be taken to avoid or reduce the harm, and that these actions need to be proportionate to the seriousness of the potential harm. In other word, in the absence of evidence take a conservative approach.

However applying the precautionary principle to EIDs in an attempt to protect the public can result in what, in retrospect, amounts to an excessive response. This occurred with attempts to control Nipah infection, where significant damage was inflicted on industry, livelihoods and the economy. Similarly, experience with highly pathogenic avian influenza (HPAI) H5N1 in China and SE-Asia showed that overzealous policy responses can destroy livelihoods and threaten food supplies [40, 41]. In Vietnam alone, almost 40 million birds were culled in 2004 in an attempt to eradicate HPAI. Although many birds were owned by large commercial operations, others were kept by ‘backyard’ farmers and villagers. Mass culling of poultry appears decisive, but places excessive burdens on vulnerable populations, is ineffective in the context of extensive ‘backyard’ poultry farming and can, in fact, promote the spread of disease [42]. A similar scenario is currently playing out with Rabies control in Bali.

Unfortunately, the precautionary principle and analytic tools and concepts appealed to in this domain, fail to deliver what is required at times of EID outbreaks since they do not advance public engagement or help resolve disagreements in times of uncertainty [43, 44]. Philosophical critiques of the precautionary principle applied to EIDs have also shown its limitations, including that defining criteria by which to judge a threat as *plausible* and a response *proportionate,* often will only substitute one uncertainty for two others [45].

(2) **Ethical challenges**

The effectiveness of an EID control policy will depend on the context of its implementation and particularly its alignment with stakeholder and public values [17, 46]. In modern liberal democracies at least some consensus over what is in the public interest and an understanding of the values which support it, is therefore required for the successful implementation of EID responses. Yet this is precisely what has been lacking in outbreaks where fracture lines, differences and value conflicts have become apparent. When the stakes are high, evidence and the implications of actions are uncertain, the situation is complex and resources may be limited but where decisions need to be made, differences are exposed. Such differences could be around beliefs about how to deal with ecological and environmental issues, which may conflict with the importance people attach to public goods, protection of individual autonomy and animal welfare [47]. These conditions of crisis and division are conducive to undesirable consequences including public fear, mistrust, misinformation and non-compliance with public health directives. For example in Canada during the SARS crisis, leaders were unprepared for the range of ethical conflicts that arose, including those over: individual freedom versus the common good; healthcare workers’ safety versus their duty to care for the sick; and economic costs versus the need for containment [48]. As indicated in Box 2, both the outbreak itself and fear that another outbreak could occur had significant economic consequences.

Any approach which hopes to successfully respond to EID threats, including a One Health approach, needs to address the ethical concerns articulated above. To this end, potentially conflicting values and logic must be negotiated to realise effective, sustainable and just solutions. Prioritisation and resource allocation require political processes based on fundamental ethical questions about what is valuable, what is to be protected and, ultimately, what is dispensable. To be effective, public policy must be consistent with the values of citizens to whom it is applied, otherwise it can become mired in controversy about whose values should prevail [31, 37, 49]. Therefore, one of the first and most important tasks of policy work is to establish how the public interest is best defined.

(3) **Legal challenges**

The legal environment in which EID policy is made and in which responses to outbreaks occur, presents its own set of challenges. The law surrounding EID responses in most jurisdictions is diffuse, complicated and often subject to re-interpretation on the basis of whose interests are given primacy at the time decisions are made. Moreover, in many countries different approaches by State/Provincial and local authorities, overlaid by Federal/National powers, complicate regulation so much that ‘hard law’ is often replaced by resort to ‘soft law’ of executive and administrative powers and international instruments, such as the International Health Regulations (IHR) [50]. This may add complexity and confusion to the EID regulatory structures, rather than facilitating public health responses to a new threat. Such confusion provides a salient reminder that even in ‘global law’ approaches to EIDs, the sovereign state remains the institution responsible for regulation and control [51].

Public health law responses to EIDs tend to be oriented towards controlling cross-border pathogen transfer and community outbreaks rather than the underlying deficiencies and structural conditions from which the threats emerge. Other laws, such as environmental law, may be more useful in addressing structural conditions for emergence. Changes in land use and agricultural intensification in developing societies are major drivers of EID. However, the cost of laws that restrict development may be greater global health inequities, with consequential effects for health outcomes. In order to clarify EID-related legal tensions between economic development and health security, a more explicit recognition is needed of who are the primary beneficiaries and who bears the costs of a One Health approach to EIDs [52].

Legal clarity around the frameworks designed to protect populations from EIDs is critical to providing an enabling infrastructure to co-ordinate and support the One Health-based work of policymakers, development planners, human and animal health-workers and biosecurity agencies.

## Discussion

### One health and public values: implications for EID control and prevention

The health of humans, animals, and ecosystems are interconnected. A One Health approach promises a better understanding of how to prevent and control EIDs at the human-animal-ecosystem interface. However the socio-political, ethical and legal challenges of EIDs illustrated above highlight how responses to infectious disease threats are intrinsically value laden. When a new infectious pathogen such as Hendra or Nipah virus first appears, or a known threat such as Rabies or Ebola encroaches on a new setting, there is limited scientific evidence or past experience to guide decisions or determine whether a planned response will be proportionate. Vastly different interpretations of EID events and their likely outcomes might be supported by the available data. Policymakers and practitioners therefore have little guidance as to what they should do when faced with a nascent infectious disease threat, only what they can do. As others [52–54] have cogently argued, they must therefore ask themselves: whose *health* is being prioritized; which *public* and which *good* are we seeking to protect?

Notwithstanding recognition of a need for complementary work on values-based questions that inevitably surround EID risks and EID control, the adoption of the One Health approach, so far, has not included development of a comprehensive, ethically-informed policy and implementation framework; this has limited its practical utility [9, 55]. Despite rhetorical and some financial support for One Health as the guiding ethos by which to address interconnected human, animal and environmental health issues, its impact will be minimal unless implications of uncertainty on, and potential conflicts between, human values and political processes are recognised and articulated. Any attempt to address these ethical and normative dimensions must take into account the dynamic nature of EID risk management. A policy that seems reasonable today may be inappropriate tomorrow, in light of new evidence. And when situations are uncertain, decision-makers inevitably fall back on their values. Therefore, a solid framework based on shared values is needed to support decision-making surrounding EIDs when “evidence” is – or may be – unreliable, and rapidly changing or fluid.

### What is needed to guide a one health approach to EIDs?

To successfully meet the challenges described above, particularly the necessity to align EID policy with public values, a One Health approach needs to engage in the following.

(i) Social science and economic research to help catalogue and describe the drivers, mechanisms and social and political configurations through which EIDs become threats to human, animal and ecological health [56, 57]. The complex connections between individual social needs and the local socioeconomic context of affected or at-risk communities, need to be understood and addressed by policymaking processes. This should ensure that manifest injustice, livelihood-based decisions and other social and cultural factors do not undermine the effectiveness of favoured control measures. Without adequate knowledge of specific local arrangements, there is a danger that insufficiently nuanced or unified approaches to EIDs will actually undermine the heterogeneous relationships and contingent practices that make health possible in circumstances of structural disadvantage [56, 58].

The social sciences are analytically broader and more policy focussed than the natural sciences. Whereas the natural sciences tend to frame infectious disease threats narrowly as matters of biological integrity and security, such that barrier technologies and hygiene practices dominate the logic of interventions [59], social science approaches go beyond this. Building social scientific evidence for use in conjunction with natural scientific evidence about EIDs aligns with the growing realization that EID emergence is as much about the social and economic configuration of capital flow as it is about the biological features of host-pathogen interactions. Current approaches to the economic and structural drivers of EID emergence still presume that state and market neoliberalism is part of the natural order, even as evidence is mounting that these systems of development are central to the problem [60, 61]. Moreover, the current emphasis on microbiology and focus on newer molecular techniques to characterise pathogens, is drawing attention away from developing better understandings of the environmental, economic and social drivers of EIDs. While this is understandable given the desire for vaccines and drugs to solve EIDs, if One Health researchers and practitioners broaden their approach to causality to include upstream, social and economic systemic causes, questions and issues that have been traditionally bracketed or thought best avoided will become central to the cross-sectoral collaboration implied by One Health.

(ii) The development of a *One Health Analytic Framework* (OHAF) needs to be pursued. Such a framework would catalogue case-based experiences and reflect the particular dynamics of specific EIDs, and promote inter-sectoral collaboration and knowledge synthesis, including integration of information about social, cultural and economic impacts, control measures and uncertainty [62, 63]. The framework would serve as a prompt to ensure that minority perspectives are represented and all relevant concerns are considered. An OHAF could provide a rubric for comparisons between outbreaks. This would allow the inherent complexities of economic and societal responses to EIDs to be compared, to inform policy processes. It is vital for discussions about EID prevention and control to have this kind of sound empirical foundation, because uncertainty and media coverage have the potential to drive bad policy.

Development of an OHAF could be facilitated by adopting well established and methodically rigorous processes such as *Framework Analysis,* produced by the National Centre for Social Research (UK) [19], or *Multi-Criteria Decision Analysis* [MCDA] developed within the field of decision science [64]. In the first instance Framework Analysis would allow for systematic incorporation of the perspectives and contributions of different scholarly disciplines and expert stakeholders. Framework Analysis facilitates movement between different datasets, thematic areas, theoretical resources, and levels of abstraction without loss of conceptual clarity [65]. The Framework method is used to organize and manage research and interpretation through the process of summarization, which is codified into a robust and flexible matrix that allows the policymaker/researcher to analyze data both by case and theme. It is commonly used in areas such as health research, policy development and program evaluation. Equally, MCDA methods offer an alternative and potentially complementary approach to OHAF development. Comprised of a suite of analytic strategies, MCDA have been shown to be valuable tools for prioritization and decision-making in animal and human health [64]. MCDA provides a framework to compare policy alternatives with diverse and often intangible impacts, which can be particularly useful in determining and justifying the prioritization and mobilization of limited research and public health resources [66, 67].

(iii) Genuine processes of public engagement across the developed and developing world are also essential to a successful One Health approach. These processes are not so much about engaging in deliberative democracy for policy decision-making, as about defining the principles and values that should guide decision-making. This means procedural inclusiveness alone is not enough to ensure transparency and reflexivity, to capture people’s preferences and to effectively communicate with the public [68].

The successful implementation of the One Health approach to EIDs will depend on public trust and co-operation. Public support for unpalatable measures is more likely if citizens understand the issues, and policy implementation reflects community values and preferences. To this end, citizens’ juries have been employed in the UK, Australia, the US and elsewhere [69–72] to explore similar issues and identify citizens’ preferences. They represent informed public opinion better than other social research methods (e.g. surveys or focus groups) because they give participants factual information, bring them into a structured and constructive dialogue with experts, provide them with time to reflect and deliberate, and allow them to represent their views directly to policymakers.

(iv) The development of a clear *Statement of Principles and Values* (SPV) for integration of ethical principles and values into decision-making is needed. This should be based on empirical data about people’s beliefs, including which public health outcomes and public goods should be prioritized and why; how to adjudicate conflicting claims and preferences; and what levels and types of evidence should be privileged in these decisions.

To be successful, One Health needs to be about more than disease prevention and control. The dynamic, unpredictable effects and risks to peoples’ lives of EIDs necessitate a public health and biosecurity infrastructure equipped to address the ethical problems that arise. EID management must therefore be based on normative principles as well as local knowledge, operational experience and disease-specific scientific and economic evidence. This means that governments and policy-makers need to explain and justify the values that underlie decision-making and engage the public in discussions about ethical choices, so that when difficult decisions arise in the face of uncertainty, they will be accepted as fair and essential for the public good [47]. This necessitates that the guiding values and likely ethical choices need to be articulated in a formal statement in advance, as in the heat of emerging health threat, decision makers will be under pressure from many sources to ‘do something quickly’.

(v) Integration of an OHAF and SPV with the IHR and relevant national health and biosecurity legislation is essential so that policymakers and practitioners can dynamically test their decision-making.

Our response should of course be based on the best scientific evidence, but EIDs are not just scientific issues, they also have significant social, ethical and animal rights dimensions. Experiences of infectious disease threats such as BSE/vCJD and SARS indicate that there have been problems combining evidence and human values at both local and policy levels [28, 73]. The communicability of diseases between species raises social, ethical and legal issues that have not been clearly elucidated or adequately addressed. Our response to nonhuman animal disease is not determined solely by bio-scientific knowledge; the way people and animals live with and amongst each other is also shaped by social norms, economic imperatives and human values. In matters of public health it is no longer sufficient to ask what works and what is the strength of the evidence; we also need to ask ethical questions about how we should seek to live, and what is the right thing to do [74–76]. Consensus about the best approaches to EID control and prevention are not always possible, however an agreed set of guiding principles and values can be a means to ensure dialogue, if not always agreement.

The development of an OHAF and SPV will also promote clearer communication about public risk. Significant EID threats have major implications for distribution of scarce resources, access to and regulation of health services and maintenance of social order. As described above it is also clear that policy and legal responses to EID threats are often highly politicised and compromised by failure to communicate clearly with the public. Policymakers responsible for responding to disasters such as EIDs typically find that there is a dissonance between transparency that may appear alarmist versus withholding information to avoid panic. Regardless of advice, people will make their own decisions based on their interpretation of available information, from formal and informal channels. So public communication, before and during a public health emergency, is frequently as important as political decisions and regulatory changes [39, 77]. This means that, to be effective, a One Health approach – like any EID policy – must deal with scientific uncertainty, whilst addressing the socio-political, ethical and legal dimensions of effective health communication and intervention strategies [3]. By exposing decision-making processes to reveal the scientific and normative uncertainties and ethical complexities, the introduction of an OHAF and a SPV into One Health theory and practice may incorporate iterative deliberation and learning into EID policy processes.

(vi) Finally, One Health must be about genuine reform rather than merely rhetoric. A One Health approach rests on the assumption that the cross-sectoral integration of expertise, research methodologies and public health infrastructure will inevitably improve capacity for disease-risk prediction and effective intervention. However, calls for increased inter-sectoral co-operation by public health practitioners, clinicians, scientists and policy-makers are not a new phenomenon. For example in the 1990s advocates of *“new public health”* called for health authorities to turn their attention to the social, economic and environmental factors that affect health – requiring the realignment and policy integration of Health Departments with other government agencies [78, 79]. Unfortunately in this case as others, attempts at promoting inter-sectoral approaches rarely move beyond rhetoric – even when driven by the best intentions and supported by substantial resources [80–82].

The problem is that arguments that promote the need for greater co-operation between sectors tend to focus on the likely benefits of collaboration rather than what reform would entail – that is, what needs to be done organisationally and politically to achieve the desired outcomes [83]. Established ‘sectors’ – whether orientated towards human or animal health, agriculture or the environment – have genealogies, traditions and rationalities of “what we are here for” that have been shaped by social, political and administrative processes [11]. As institutions, they are philosophically and structurally resistant to change that diverts resources and re-orients practices away from their own sectoral priorities [83]. In essence, they have their own constituencies to serve. As a consequence, establishment and implementation of mechanisms that enhance information-sharing, collaboration and inter-sectoral co-operation, such as working groups and interdepartmental committees, have rarely delivered the outcomes promised in the past. Responses to BSE/vCJD in the UK [28], HPAI in South East Asia [11], and recent case studies of One Health programs in Uganda [84], suggest that more work is needed to co-ordinate implementation and overcome sectoral interests. The complexity of the problems posed by EIDs mean that organising effective control and prevention programs will require genuine cross-sectoral integration and, potentially, re-sectoring of some institutional and professional responsibilities [62]. And as the recent Ebolavirus disease outbreak illustrates, there must also be sustained social and political willingness to achieve control.

If One Health is genuinely the way forward, as we believe it is, then we should do more than talk about its potential benefits. Without genuine cross-sectoral reform and a radical broadening of the scope of its inquiry into how specific social, cultural and spatial configurations promote the risks of EID emergence, One Health is in danger of becoming merely a rhetorical strategy to avoid conflict between its core disciplines, whereby practitioners, researchers and policymakers will espouse the methodological and moral case for interdisciplinary collaboration yet remain in their silos [11]. Even if these barriers are overcome the One Health approach will only succeed if it explicitly acknowledges local contingent and contextual dimensions of disease risk and disease expression and the political impacts of scientific uncertainty, while also seeking to accommodate the values and preferences of ‘at risk’ and affected individuals. Further, we suggest that decision making around EIDs requires an ethical framework that reflects the values of affected and ‘at risk’ communities, privileges justice, takes account of human flourishing, protects animal health and welfare and is developed in consultation with relevant stakeholders and the public.

## Conclusions

EID risk management is a major global public health issue to which One Health represents a promising approach, but its potential benefits have not been fully realised [53, 55]. Despite recognition that the social and cultural dimensions are critical to the success of One Health, social scientists are yet to play a central or substantive role in shaping research programs and interventions [12, 13]. At the same time as the literature on the ethics of pandemic responses and preparedness continues to grow, the One Health approach to EIDs has received little formal ethical consideration. Even the most ethically attuned existing frameworks for biosecurity and infection prevention and control provide only general operational principles that do not guide actions in times of uncertainty. If One Health is to be meaningful − let alone successful − more attention must be paid to how these different types of knowledge are brought together and brought to public attention. Effective responses to EIDs are likely to be delayed or precluded unless all the socio-political, ethical and legal implications are articulated, publicly debated and − as far as possible − resolved in advance. Policy makers and public health experts need a set of principles and values, developed and articulated prior to an outbreak that explicitly acknowledge the preferences of affected communities and can guide integration of new evidence into decision-making processes in a dynamic manner.
